# The Association of Emotional Eating with Overweight/Obesity, Depression, Anxiety/Stress, and Dietary Patterns: A Review of the Current Clinical Evidence

**DOI:** 10.3390/nu15051173

**Published:** 2023-02-26

**Authors:** Antonios Dakanalis, Maria Mentzelou, Souzana K. Papadopoulou, Dimitrios Papandreou, Maria Spanoudaki, Georgios K. Vasios, Eleni Pavlidou, Maria Mantzorou, Constantinos Giaginis

**Affiliations:** 1 School of Medicine and Surgery, University of Milano-Bicocca, 20900 Monza, Italy; 2 Fondazione IRCCS San Gerardo dei Tintori, 20900 Monza, Italy; 3 Department of Food Science and Nutrition, School of Environment, University of Aegean, 81400 Myrina, Greece; 4 Department of Nutritional Sciences and Dietetics, School of Health Sciences, International Hellenic University, 57400 Thessaloniki, Greece; 5 Department of Health Sciences, College of Natural and Health Sciences, Zayed University, Abu Dhabi P.O. Box 144534, United Arab Emirates; 6 Clinical Dietetics & Nutrition Department of 424 General Military Hospital, New Efkarpia Ring Road, 56429 Thessaloniki, Greece

**Keywords:** emotional eating, overweight, obesity, depression, anxiety, stress, dietary patterns, nutrition, mental health

## Abstract

(1) Background: Emotional eating is considered as the propensity to eat in response to emotions. It is considered as a critical risk factor for recurrent weight gain. Such overeating is able to affect general health due to excess energy intake and mental health. So far, there is still considerable controversy on the effect of the emotional eating concept. The objective of this study is to summarize and evaluate the interconnections among emotional eating and overweight/obesity, depression, anxiety/stress, and dietary patterns; (2) Methods: This is a thorough review of the reported associations among emotional eating and overweight/obesity, depression, anxiety/stress, and dietary patterns. We compressively searched the most precise scientific online databases, e.g., PubMed, Scopus, Web of Science and Google Scholar to obtain the most up-to-date data from clinical studies in humans from the last ten years (2013–2023) using critical and representative keywords. Several inclusion and exclusion criteria were applied for scrutinizing only longitudinal, cross-sectional, descriptive, and prospective clinical studies in Caucasian populations; (3) Results: The currently available findings suggest that overeating/obesity and unhealthy eating behaviors (e.g., fast food consumption) are associated with emotional eating. Moreover, the increase in depressive symptoms seems to be related with more emotional eating. Psychological distress is also related with a greater risk for emotional eating. However, the most common limitations are the small sample size and their lack of diversity. In addition, a cross-sectional study was performed in the majority of them; (4) Conclusions: Finding coping mechanisms for the negative emotions and nutrition education can prevent the prevalence of emotional eating. Future studies should further explain the underlying mechanisms of the interconnections among emotional eating and overweight/obesity, depression, anxiety/stress, and dietary patterns.

## 1. Introduction

Obesity has grown into a major public health issue worldwide. Obesity increases the risk of several diseases, including diabetes type 2, high blood pressure and cholesterol, musculoskeletal issues, and several types of cancer [1]. The factors that render individuals prone to overeating have been investigated comprehensively. Recent evidence suggests that there is an association between psychological factors and the etiology of obesity [2]. The existing literature research suggests that deficits in emotion dysregulation and a high level of negative emotions are crucial in the progression and prolongation of obesity [3]. Thus, there is a bidirectional correlation: obesity could not only result in physical diseases, but also occur in addition to psychological disorders and social problems, such as low self-esteem, depression, and social stigma [4]. 

Moreover, stress and emotional conditions affect the eating behavior [5]. Stress and negative mood are able to influence appetite inversely, prompting several people to eat more and others to eat less [6,7]. These behaviors, known as emotional overeating and emotional undereating, have been associated to the beginning of body weight complications and eating disorders [8,9]. Nevertheless, concerning a significant subgroup of people of both sexes, negative emotions, and stress render them to overeat—a kind of eating known as emotional eating [10,11]. In addition, abnormal emotional functioning means difficulty in appropriately recognizing, understanding, and coping with emotions and a long-term experience of elevated levels of harmful emotions [3]. Notably, elevated negative affectivity, body dissatisfaction, self-objectification, and lower self-esteem were predictive of persistent engagement in regular binge eating and inappropriate weight compensatory behaviors [8,9]. Self-objectification (thinking and monitoring the body’s outward appearance from a third-person perspective) emerged as the largest contributor of both the initiation and persistence of all behavioral symptoms [8,9].

Emotional eating is not a separate eating disorder, but an eating behavior that is influenced by behaviors, stress, emotions, and individual feelings in relation to eating. However, it must be clear that emotional eating, unlike specific eating disorders, is not related with a total failure of keeping under control the quantity and quality of food consumed [12]. Emotional or comfort eating, as well as stress-induced eating, leads to the predisposition to eat in response to negative emotions, with the preferred foods being mainly energy-dense, poor in nutrients and tasty [13,14,15]. These types of eating act as a coping mechanism to control and decrease negative emotions, such as depressed mood, anxiety, and stress [16]. As it concerns the prevalence of emotional eating, based on the Stress survey in America performed by the American Psychological Association, 38% of adults stated that they have been implicated in emotional eating during the past month, with 49% of them engaging in it weekly [17]. Furthermore, it is frequently considered as a main factor in models of eating disorders and pathological eating attitudes such as overeating and binge eating [9,18], and could lead to substantial psychological distress and health issues [19]. Hence, it is important to interpret its fundamental mechanisms [16]. 

Emotional eating has a positive relationship with increase in weight gain over time and difficulty of losing weight. These can be attributed to the fact that emotional eaters are more prone to greater consumption of sugary and high-fat foods, eat in response to stressors, and snack more frequently compared to non-emotional eaters [20]. Notably, a cross-sectional analysis of the NutriNet-Santé cohort conducted on 7378 men and 22,862 women reported that greater emotional eating was related with elevated consumption of energy-dense snacks, such as sweet and fatty foods, and these associations were predominantly stronger in women suffering from depression [21]. Moreover, an association between excessive alcohol consumption and emotional eating (binge eating disorder) has been reported [22]. These eating habits combined with increased body weight put emotional eaters at a higher risk of diabetes and heart disease [23]. Furthermore, emotional eaters activate the relationship of eating motive and reward, supporting evidence that food exerts a reward effect and therefore can alleviate or lessen negative mood conditions [13]. In addition, emotional eaters frequently feel negative emotions concerning their physical appearance right after the eating events. Practically, every person has experienced the impact of emotions on eating attitudes. On the contrary, eating behavior is also able to affect influence body image, resulting in body dissatisfaction, which implies to the body-related negative self-assessment of a person [17]. 

Considering the above, the aim of the present study was to provide a thorough review of the reported interrelationships among emotional eating and overweight/obesity, depression, anxiety/stress, and eating patterns established in clinical studies at the last decade. 

## 2. Methods

Thorough research of the current international literature has been performed for the last ten years (2013–2023) in the most precise scientific databases, e.g., PubMed, Scopus, Web of Science and Google Scholar, using critical and representative keywords such as emotional eating, overeating, psychological disorders, binge eating, depression, anxiety, stress, obesity, overweight, weight gain, body mass index, appetite, etc. Inclusion criteria were longitudinal, cross-sectional, descriptive, prospective clinical studies with Caucasian participants. Exclusion criteria were all studies written in a language other than English, as well as studies involving animals. Review articles, case report studies, commentaries and abstracts in congress proceedings were excluded. The results were filtered according to relevance and the most relevant ones were selected and quoted below. Only clinical human studies with a strong protocol design that applied a validated questionnaire to diagnose emotional eating were included in this review. The retrieved studies were further reviewed for related studies cited in their text. 

## 3. Results and Discussion

### 3.1. Emotional Eating and Obesity

Obesity is a serious threat to human health, and its prevalence has almost tripled since 1975. Moreover, the etiology of obesity is multifactorial and eating behavior exerts a crucial role [2]. Stigma and weight prejudice are associated with psychological issues and unhealthy eating behaviors (such as emotional eating). The main findings of the studies that examined the relationship between emotional eating and obesity are presented in Table 1. 

A case–control analytic survey by Varela et al. examined the association among coping approaches, eating behaviors and Body Mass Index (BMI) in 473 participants with the use of Dutch Eating Behavior Questionnaire (DEBQ) [24]. The research demonstrated that overweight individuals scored considerably higher in emotional eating compared to normal-weight individuals. A strength of this study was the intention to evaluate both coping approaches and eating behaviors as well as their association with BMI. These results supported the findings of earlier studies that also noted and further documented these differences [24]. There are also some limitations to consider in this study, such as the higher proportion of women and self-reported data on height and weight. However, due to the complex behaviors associated with weight gain and weight management, it is essential to utilize a less critical and more open-minded attitude to design effective treatments [24]. 

Additionally, Madali et al. evaluated emotional eating predisposition of Turkish individuals during COVID-19 pandemic from August to September 2020 with the use of Emotional Eating Scale (EES) [25]. They determined that emotional eating was more frequent in obese people, while most of the individuals (75.7%) were emotional eaters [25]. Due to the COVID-19 measures, a significant alteration in the lifestyle and behaviors of the individuals had reasonably occurred. However, it should be noted that this study has some limitations. In fact, the data were compiled by an online questionnaire and its results should not be considered as representative for the Turkish population [25]. Moreover, body weight and height information were self-reported and thus they were not measured by specialist physicians or nutritionists. Notably, as the pandemic is continuing, additional investigation of this issue is strongly recommended [25].

Tatjana van Strien et al. investigated whether three psychological eating patterns (emotional eating, external eating, and restrained eating) play a role as mediators between depressive behavior and weight increase in 592 adults using self-reported questionnaires: DEBQ and depressive mood list (DML) [26]. They also evaluated the balance impact of 5-5 HTTLPR genotype (greater risk for depression) in a sub-group of 520 Caucasians [26]. This study provided evidence that emotional eating may act as mediator between depressive symptoms and future body weight increase in women and suggested that this mediation influence could be depended on 5-HTTLPR genotype. A strength of this study was the large sample size used, which provided this longitudinal study with substantial power [26]. On the other hand, the main limitation of this study was the difference in frequencies in 5-HTTLPR allele among ethnicities. Therefore, these results could not be generalized to other human populations, except for Caucasians. A limitation of this study concerned the usage of self-reported questionnaires. Furthermore, given that the study sample was non-clinical adults, research needs to consider whether these results can be applied to clinical subjects [26].

Furthermore, a cross-sectional study assessed the associations among emotion dysregulation, psychological distress, emotional eating, and BMI in 600 Italian young adults aged 20–35 years [27]. The participants completed demographical and physical data, the Difficulties in Emotion Regulation Scale (DERS), the Depression Anxiety and Stress Scale (DASS-21), and the Emotional eating subscale of DEBQ via an online anonymous survey. It was determined that emotion dysregulation contributed to elevated psychological distress and emotional eating levels that, in turn, were associated with greater BMI [27]. However, this study was also not free of limitations. Firstly, the cross-sectional kind of this study does not support causality. Secondly, due to the use of a convenience sample of young adults, the findings could not be extrapolated to the general population. One more limitation deals with the absence of clinical instruments that evaluate potential eating disorders among individuals. Overall, this study advocates for improving psychological mediations intended to encourage emotional management approaches and directed at promoting physical and psychological wellness [27]. 

Additionally, a prospective study of Marc Bénard showed a positive relation between emotional eating and BMI in 39.771 adult men and women [28]. Participants completed three questionnaires: the revised 21-item Three-Factor Eating Questionnaire (TFEQ-R21) for the evaluation of eating behaviors, (CFC-12) questionnaire for the evaluation of consideration of future consequences and the Barratt Impulsiveness Scale (BIS-11) questionnaire for the examination of impulsivity [28]. The findings showed that emotional eating was positively related with BMI in both men and women, with the latter demonstrating a greater association. Consideration of future consequences and impulsivity quantitatively attenuated the relationship between emotional eating and BMI but did not affect the link between emotional eating and BMI shift [28]. The relationship between emotional eating and BMI was greater in women and men with a low future orientation, and in women presenting an elevated impulsivity level. The main strength of this study was its large sample size that permitted the usage of several covariables for adjustment for potential confounders [28]. In addition, it presents new prospects to better recognize emotional eating and its relationship with body weight. On the other hand, the main limitation of this study concerned the delay among the completion of the TFEQ-R21 and the CFC-12 and BIS-11. Other limitations include self-reported anthropometric measurements and the selection bias due to the procedure employed to enroll individuals that was established by volunteers. In this aspect, interventional strategies targeting cognitive control may be assessed for their capability to enhance consideration of future consequences and/or reduce impulsivity in association with eating attitudes [28].

Kamila Czepczor-Bernat et al. evaluated the influence of food-related behaviors and emotional performance on BMI in 298 adults [29]. All of them completed the DERS, the Positive and Negative Affect Schedule (PANAS), the Feeling of Stress Questionnaire (FSQ) as well as the TFEQ-R18. This study showed that stress, negative emotions, and emotional dysregulation had an indirect effect on BMI [29]. Thus, the positive and direct connection between emotional eating and BMI could support evidence that decrease in emotional eating could result in reduced BMI. However, this study had some limitations. The design of the study was cross-sectional, and the number of participants was small. In agreement with the previous studies, this study suggested that future interventions should be focused on reducing body mass by modifying the necessary eating behaviors with emphasis on the patient’s emotional status and improvements in adaptive skills in order for emotional modulation to be improved efficiently [29]. 

Furthermore, Hedvig Sultson et al. investigated the phenotypes of women presenting emotional eating according to their positive and negative emotional eating, BMI and preoccupation with body weight levels [30]. This study also assessed possible differences in eating pathologies and emotional modulation complications among them. The sample was 605 women who completed the Positive–Negative Emotional Eating Scale (PNEES), Eating Disorders Assessment Scale (EDAS), and DERS [30]. The researchers showed four-profile subtypes that demonstrated differences in various sights of emotion regulation and eating disease psychopathology. More to the point, the four profiles were the following: obese individuals with high levels of emotional eating (because of positive and negative emotions), normal-weight women with low levels of emotional eating (because of positive and negative emotions), overweight persons without emotional eating and normal-weight persons with emotional eating [30]. This study highlighted that the existence of an enhanced level of negative emotional eating among the profiles may be a highly significant sign of eating disorder psychopathology, while high BMI itself was not. However, this study has certain limitations. Firstly, the size of groups was different, and this may affect the findings of the pairwise comparisons. Secondly, causality could not be proven due to the cross-sectional design of the study. Undoubtedly, future investigations are recommended to assess the association between emotional eating and emotional dysregulation [30]. 

A prospective study performed by Konttinen et al. (n = 3735) evaluated the role of emotional eating in the relationship among depression, BMI and waist circumference [31]. The participants completed the 20-item Center for Epidemiological Studies—Depression (CES-D) Scale, TFEQ-R18, questions for the assessment of night sleep duration and the International Physical Activity Questionnaire—Short Form (IPAQ-SF) [31]. This study showed that eating due to negative emotions facilitated the positive relationships among depression and rise in BMI and waist circumference over 7 years. Night sleep duration attenuated the correlations of emotional eating, too [31]. The main strength of this study was its prospective design with a large population-based sample. In addition, it provided new perceptions on depression and emotional eating as risk factors for (abdominal) obesity. Nevertheless, some limitations are required to be considered while interpreting the above findings, such as self-reported data, the restrictions of CES-D scale and TFEQ-R18. In this aspect, future studies need to evaluate the clinical significance of the study findings [31].

Additionally, in the study of Irina Lazarevich et al. [2], the relationship between emotional eating and BMI in nonclinical sample was confirmed. This study evaluated the relationship among depressive symptomatology, emotional eating, and BMI in Mexican college students (n = 1453) and assessed emotional eating as a mediating variable between depression and obesity [2]. Participants filled out the scale of Self-Efficacy in Emotion- and Stress-Related Eating (SEESE) to evaluate emotional eating and the CES-D to measure depression symptomatology [2]. Significant associations between depression symptoms and emotional eating, as well as between emotional eating and BMI, were noted. Additionally, emotional eating was identified as a mediator between depression and BMI [2]. The indirect impact of depression due to emotional eating on BMI constituted a significant proportion of the overall impact in men (23.1%) and women (25.0%). One of the advantages of this study was that both measured weight and height data were used in every individual that provided more precise information compared to self-reported methods, also highlighting the novel evidence of the regulating impact of emotional eating in the association between depression symptomatology and BMI in a young, educated population [2]. Regarding the main limitations of this study, those were the specific non-clinical student population and the self-reported data in questionnaires. Thus, longitudinal studies are strongly required to assess the long-term impact of depression and emotional eating on body weight [2].

Lorena S Pacheco et al. investigated the relationship between eating behavior scores (cognitive restraint, uncontrolled eating, and emotional eating) and body composition in 555 Chilean young adults [32]. The participants completed the TFEQ-R18. In addition, their body fat percentage was measured. This study indicated that elevated emotional eating scores were more frequently observed in women compared to men. In addition, an elevated risk of emotional eating was detected among obese adults compared to those without obesity, independently of the obesity measures used. This study had several advantages [32]. The data were highly reliable as all anthropometry, adiposity measurements and eating behavior measurements were compiled at an academic nutrition research institute by well-qualified personnel based on standardized methods. Moreover, body composition was evaluated by dual-energy X-ray absorptiometry (DXA). Concerning limitations, the cross-sectional nature of the study and the self-reported data in the completion of questionnaire did not provide causality [32]. Hence, further investigations addressing the longitudinal associations between eating behavior and body composition are strongly recommended. 

In a recent cross-sectional study performed in 400 young Peruvian adults, emotional eating was more frequent in overweight and obese participants compared to those with normal weight and underweight [33]. However, in this study, anthropometric data were self-reported and not measured [33]. Another recent study conducted in Polish adolescents also reported that a greater part of emotional eaters and very emotional eaters were found among overweight and obese participants compared to the other groups in the period of the COVID-19 pandemic from 21 January 2021 to 17 February 2021 [34]. In this study, female participants, obese participants, and those experiencing weight gain were especially more susceptible to emotional eating behaviors compared to others, highlighting that suitable education needs to be provided to susceptible individuals such as female and obese adolescents [34]. However, this study had several limitations, since it was an online survey with cross-sectional design, which used self-reported anthropometric data to calculate participants’ BMI [34].

### 3.2. Emotional Eating and Depression

Depression is a heterogeneous syndrome that is gradually increasing in general population at an alarming rate. It raises the risk of several chronic disorders, including cardiovascular disease (CVD), diabetes mellitus and body weight gain over time. In addition, it contributes significantly to the financial burden and disability of the public [35]. Depression is characterized by repeated, severe, and overwhelming negative emotions and impacts [36]. 

Moreover, it is also characterized by appetite loss and subsequent weight reduction, yet there is also a subtype of depression characterized by the atypical vegetative symptomatology of an elevated appetite and increase in body weight. Emotional eating has been proposed as an indicator of atypical depression, because it shares with this depression subtype the atypical feature of enhanced appetite in response to distress [37]. The clinical studies that examined the association between emotional eating and depression are presented in Table 2.

As mentioned above, the studies of Tatjana van Strien et al., Irina Lazarevich et al., and Hanna Konttinen et al. demonstrated that depression was associated with increased emotional eating [2,26,31]. Additionally, the cross-sectional study of Braden et al. simultaneously examined psychosocial associations of eating in response to depressive symptoms, anxiety/anger, boredom, and positive emotions (N = 189) [20]. The EES and the Emotional Appetite Questionnaire (EMAQ), which were helpful for the examination of eating behaviors, were implied. A broad variety of psychological symptoms was examined by Symptom Checklist-90-Revised (SCL-90) [20]. The Eating Disorders Examination Questionnaire (EDE-Q) was applied for eating disorder diagnosis. The DERS was also used, which is a 36-item self-report measure intended to assess complications in several aspects of emotion regulation. Finally, the short-form health survey (SF-12) was completed to examine general physical health [20]. In this group of adults affected by overweight/obesity, eating caused by depression, anxiety/anger, and boredom was related with worse psychological well-being, more severe eating disorder symptoms, and more emotion regulation complications. Eating caused by positive emotions was not related with negative outcomes [20]. Additionally, all types of emotional eating were not associated with worse self-reported physical health. In spite of the current attention to eating due to positive emotions, eating caused by negative emotions appears to have a closer association with psychological complications [20]. Moreover, the derived unique pattern of associations provided additional evidence for the significance of considering emotional eating as a multidimensional feature. However, this study had some limitations such as a cross-sectional design and dependence on self-report questionnaires which were subject to bias [20]. In addition, multiple analyses were performed in this study, and a Bonferroni test was applied for assessing significance in multivariate analyses; however, the probability of Type I error persisted, especially for correlational and exploratory analyses. Moreover, this study enrolled a general sample of adults affected by overweight/obesity that could not be characteristic of emotionally fueled adults who are not overweight/obese or a therapy-seeking sample [20]. Overall, these findings suggested that some emotion regulation approaches could be more strongly associated with different kinds of emotional eating. In this aspect, future research studies could focus on the evaluation of whether treatment programs should target and educate particular emotion regulation approaches to diverse kinds of emotional eaters [20].

Moreover, Ti Hsu et al. evaluated the diminishing effect of trait mindfulness in the association between psychological distress and emotional eating in their cross-sectional survey [38]. The sample size was 248 adults that completed Inventory of Depression and Anxiety Symptoms (IDAS), Five Factor Mindfulness Questionnaire (FFMQ), and TFEQ-R18 questionnaires [38]. The analyses showed a positive relationship between depression and anxiety symptoms and emotional eating [38]. In addition, the findings indicated that “nonjudging” attenuated the association between depression and emotional eating [38]. This study had several strengths and limitations. More to the point, sample diversity and dealing with the new aspects in understanding between depression and emotional eating were the strengths of this survey, while self-report data and cross-sectional design were its main limitations [38].

Nadine et al. examined the relationships between subclinical depression symptomatology, previous existence of major depression symptoms, and severity of depression and eating behavior [39]. The participants (n = 990, overweight or obese) completed the DEBQ, 30-item Inventory of Depressive Symptomatology—Self Report (IDS-SR) and semiquantitative food frequency questionnaire (FFQ) [39]. The results of this research revealed that in a sample of participants with a sub-syndromic depressive symptom, those with previous major depression disease exhibited elevated levels of emotional eating [39]. In addition, enhanced depression symptoms were more frequently related with emotional eating. This study had important advantages. It provided new findings on the relationship between depression and non-healthy eating habits; it also measured body weight and body height in every individual that provided more precise information compared to self-report practices [39]. Nevertheless, the major limitation was the cross-sectional design of this study. Furthermore, the sample was a specific population with high BMI and subsyndromal depression symptomatology, so it is difficult to generalize these results to other populations. Finally, the usage of self-report questionnaires increased the risk of underreporting.

Clémence Willem et al. explored the associations among emotion dysregulation, depression and anxiety and emotional eating in obese adults [36]. The participants filled out the DEBQ, Beck Depression Inventory Short Form (BDI-SF), and Cognitive Emotion Regulation Questionnaire [36]. The findings of this study highlighted that emotion dysregulation, anxiety and depression were affected by the severity of obesity [36]. Emotion dysregulation was directly related with more emotional eating in severe obesity but not in moderate obesity, where only an indirect association was recorded [36]. This study offered several novel perspectives. Firstly, it suggested that psychotherapeutic interventions intended to decrease emotion dysregulation could be effective for obese patients [36]. Furthermore, it indicated that decreasing emotion dysregulation and related depression and anxiety may decrease the possibility of weight increase and maintenance in this population [36]. On the other hand, this study had several limitations. The major weaknesses were the use of self-reported measures, the cross-sectional study design, and the absence of several negative emotions [36]. In this aspect, forthcoming studies need to investigate whether underlying factors may be ascribed to the diverse pathways of emotional eating in moderate obese and severe obese individuals and whether these pathways could be related to the severity of obesity [36].

Calderón-Asenjo et al. performed a cross-sectional study in 400 adults and demonstrated that the proportion of participants reporting symptoms of depression was considerably elevated in those presenting negative emotional eating compared to those without such eating behavior [33]. This study evaluated depression symptomatology utilizing the Patient Health Questionnaire-2 (PHQ-2) scale [33]. 

A recent online cross-sectional study in Turkish adults assessed the prevalence and indicators of perceived depression, anxiety, stress levels and emotional eating behaviors for the duration of COVID-19 pandemic [40]. In this study, self-reported data were retrieved from December 2020 to April 2021 utilizing Google Forms web survey software and DASS-42, a self-report questionnaire. One out of every three individuals stated moderate to severe perceived DAS during the pandemic. Emotional eating was considerably associated with perceived DAS. However, this study exhibited some disadvantages such as its cross-sectional design and the absence of longitudinal follow-up. Moreover, its findings should not be attributed to the pandemic per se as their emotional status before the pandemic was not known. As Türkiye is a huge country and Internet access remains reduced in several areas, these findings should not be generalized to the entire population in Türkiye [40].

Hatice Kübra Barcın-Güzeldere et al. examined perceived stress and emotional eating behaviors during the COVID-19 pandemic partial quarantine between June and September 2020 based on healthy adults BMI levels [41]. Participants filled out a questionnaire consisting of four parts, including 11 questions about demographic variables, and 11 questions that examined nutritional behaviors, PSS-14, and Emotional Eater Questionnaire [41]. Their results supported evidence that individuals with elevated BMI were more susceptible to emotional eating. EEQ and PSS-14 scores of women were significantly higher compared to men and obese participants consumed sweetened and carbonated drinks two-fold more than other participants. However, the major disadvantages of this survey were the self-reported data and the lack of balance in sex distribution [41].

### 3.3. Emotional Eating and Anxiety/Stress

The concept of stress is directly related with higher probability of chronic health disorders and accelerated the rates of disease development. Over the past 25 years, the association between stress and eating behavior is well recognized worldwide, and many studies have indicated that stress is related with alterations in food consumption of adults [42]. Stress-stimulated eating is characterized by an enhanced consumption of energy-dense, highly tasty food for coping with stress [43]. 

However, there are various mechanisms through which such an effect could operate. Taking into consideration that stress could influence appetite by both physiological and psychological mechanisms, relaxation could exert a comparable opposite effect in both respects [43]. However, the dysregulation of bio-behavioral responses to food intake under stress has focused the highest research interest, taking into consideration the longer-term implications for physical disease risk [42]. The main findings of the studies that examined the relationship between emotional eating and stress are presented in Table 3.

Tannia Valeria Carpio-Arias et al. in their cross-sectional study examined the relationship between perceived stress and emotional eating in 2333 adults during COVID-19 pandemic from January to March 2021 [44]. Emotional eating was evaluated through questionnaire and perceived stress was evaluated through Perceived Stress Scale [44]. This study determined that individuals who displayed perceived stress had a greater probability of being emotional eaters [44]. Moreover, the participants could in particular be susceptible to body weight increase [44]. However, the main weakness of this study was the non-probability sampling, which was performed. In addition, the data were self-reported. Thus, further research is suggested to derive more accurate recommendations on this issue [44].

Laura E. Finch et al. examined whether comfort eating buffered the association between adverse life events and perceived psychological stress and whether possible stress-buffering impacts of comfort eating may be affected by the levels of depression symptoms in 2379 young adult women [45]. Questionnaires were used to assess the adverse life events, perceived stress, comfort eating and depressive symptoms. This study showed that comfort eating seemed to buffer perceived stress merely in participants without increased levels of depression symptoms [45]. However, this study had some limitations such as self-reported data and the lack of men in the sample. On the other hand, it was the first study to assess possible stress-buffering effects of comfort eating at a population health level and in response to naturally occurring stressors [45].

The study of Guerrini-Usubini et al. evaluated the effect of emotion dysregulation on emotional eating, psychological distress, and BMI in Italian young adults [27]. The DERS questionnaire was applied for evaluating emotion dysregulation, and the DASS-21 questionnaire was applied for assessing psychological distress. Emotional eating was assessed by the DEBQ-EE questionnaire [27]. This study determined that emotional eating was not merely related with worse psychological effects but also with physical ones because it was associated with higher intake of elevated energy-density food and increased BMI [27]. Nevertheless, this study presented some limitations including the cross-sectional design, the convenience sample of young adults, the absence of clinical instruments which could evaluate potential eating diseases among participants, and the self-reported data. Further research needs to examine new concepts, including another measurement approaches, and testing the same pattern in diverse subcategories [27].

Klatzkin et al. in their study investigated whether perceived life stress or cognitive restraint could raise snack consumption under stress [46]. The study population included 43 undergraduate women. Depression symptoms, uncontrolled eating, emotional eating, cognitive restraint, and BMI were measured [46]. This study indicated that perceived life stress increased the hyperphagic impacts of stress-stimulated negative affect, which could be ascribed to higher decreases in stress-stimulated negative affect upon snacking. Notably, a very important strength of this study was the design of its analyses [46]. However, it presented certain limitations, such as the inadequate diversity of the sample and the restricted BMI range. Thus, further research is strongly recommended to inform clinical approaches for persons with prolonged or perceived life stress who are in medication due to obesity-related concerns [46].

In addition, during COVID-19 period from January to February 2021, eating motives and other factors predicting emotional overeating were examined in Polish adults [47]. Participants completed the Eating Motivation Survey, the Emotional Overeating Questionnaire, a COVID-19-associated stress measure, and a socio-demographic survey. The findings demonstrated that for the duration of the COVID-19 pandemic, overeating helped individuals to cope with the quarantine and the negative emotions related with it [47]. Nevertheless, the major disadvantages of this study were its cross-sectional design and the self-reported data. Other limitations included a single question that was used to assess COVID-19-associated stress and the convenient sampling procedure that was utilized to enroll individuals. In this aspect, forthcoming studies may apply analysis of data based on age range, lifestyle variables, more comprehensive information on the COVID-19 conditions, other eating motives and the interrelationship between COVID-19-associated stress and other variables [47]. Furthermore, the frequent adoption of this eating pattern could lead to the adoption of this behavior as a dominant mechanism for triggering stressful situations.

Another study examined the primary associations between psychosocial effect of COVID-19 pandemic and disturbed eating behaviors for the duration of the first lockdown in 254 community adults in Portugal [48]. The participants completed questionnaires for the assessment of eating behavior, Coronavirus impact, depression, anxiety and stress and emotional eating one week after the mandatory 46-day COVID-19 lockdown in Portugal (18 March to 3 May 2020). The relationship between the psychosocial effects of COVID-19 and eating disorders (emotional and uncontrolled eating) appeared to be better explained by the psychological distress experienced (depression, anxiety and stress) for the duration of the first lockdown [48]. Interestingly, the main advantage of this study was the new findings during COVID-19 pandemic and the early effects on eating behavior in Portugal [48]. However, an absence of variety in the examined population was observed, as well as a cross-sectional design. Future research should be performed to assess these variables longitudinally to prove causality in high-educated women with decreased overrepresentation [48].

Emotional eating has been connected to plain sugars consumption. Assessment of the incidence of emotional eating and its correlation with anxiety and psychological distress within the COVID-19 lockdown by a large-scale population-based survey (n = 24,968) revealed that psychological distress was related with emotional eating and greater intake of high-sugar foods and beverages [49]. Emotional eating was noted in 54% of the study population and was considerably more frequent in female individuals. This study was carried out between 15 April and 30 April after approximately six weeks of interventions to tackle the first wave of the COVID-19 pandemic. The major advantage of this survey was its large sample size, and its main limitations were the cross-sectional design and the self-reported data [49]. 

Calderón-Asenjo et al. also discovered that adults presenting anxiety symptoms were more likely to exhibit negative emotional eating than those without anxiety symptomatology. In this study, the Generalized Anxiety Disorder Scale (GAD-2) was used to assess anxiety symptomatology, which constitutes a basic criterion to diagnose anxiety [33]. Accordingly, Kaner et al. also discovered that negative emotional eating was related with the occurrence of anxiety and stress symptoms evaluated by Depression, Anxiety, Stress Scale (DASS-42) during COVID-19 pandemic [40].

A recent cross-sectional online study was performed in Turkey during COVID-19 pandemic between 15 February and 30 April 2021 [50]. In this study, elevated emotional eating increased helpless approach, submissive approach, and Coronavirus anxiety rise while reducing the self-confident approach. BMI, weight change in pandemic, age, self-confident approach to coping with stress and helpless approach score explained emotional eating at a percentage of 30.8%. However, this study had several disadvantages. One of the disadvantages is that this survey was based on participants’ self-reports, while its cross-sectional design cannot support causality [50].

### 3.4. Emotional Eating and Dietary Patterns

Emotional eating has been associated with non-healthy eating motives in which individuals eat high amounts of hyper-tasty energy-dense foods that contain high fat and sugar levels. This pattern has been observed in both men and women at diverse periods of their life. Both positive and negative emotions seem to exert a crucial influence in the selection, purchase, and intake of food [51]. Eating changes experienced in situations or events of daily routine are usually pointed to increase the consumption of foods with poor content of nutrients. It should be highlighted that the intake of such foods can substitute regular meals, subsequently leading to incorporation of such foods into the everyday diet of people [52]. The results of the available clinical studies evaluating the association of emotional eating with dietary patterns are presented in Table 4.

Alejandra Betancourt-Núñez et al. analyzed whether emotional eating was associated with specific dietary patterns in people with and without obesity [53]. After the assessment of emotional eating and nutrition habits, the findings showed that individuals who were emotional eaters and exhibited abdominal obesity followed a dietary pattern with many snacking and fast food more strongly [53]. Notably, the major advantage of this study was the completion of a validated semiquantitative food frequency questionnaire [53]. On the other hand, the main limitations were the cross-sectional design and the risk of underreport. Novel studies need to be conducted during post-pandemic period in which emotions and dietary patterns could be significantly modified [53].

In another study, Marchena Carlos et al. evaluated the eating behaviors, alcohol consumption, levels of emotional eating, and anxiety (state/trait) between students of physiotherapy, psychology, and nursing [54]. The participants filled out the Mediterranean diet (KIDMED) adherence test, the Alcohol Use Disorder Identification Test (AUDIT), the State-Trait Anxiety Inventory (STAI) and the EEQ [54]. This study reported low Mediterranean diet adherence levels among university students (15.5%) and significant levels of emotional eating (29%) and anxiety (23.6%) [54]. Nevertheless, this study had several limitations. Firstly, a more representative sample should be recruited, and secondly, the findings should be compared with students from non-healthcare courses. Future studies should focus on the design and application of learning courses to encourage healthy behaviors amongst university students [54].

As discussed before, Madali et al. evaluated emotional eating incidence of Turkish people for the duration of COVID-19 pandemic with the use of EES [25]. Concerning eating behavior, it was determined that individuals stated a rise in the intake of fresh fruit and vegetables, protein sources such as eggs, milk, and red meat, while they reported a reduction in the intake of junk food such as biscuits, chips, chocolates, and carbohydrates such as pastries, syrupy desserts, and bread due to the plethora of easily available fresh vegetables and fruit [25]. 

A recent cross-sectional survey explored the relationships between emotional eating and behavioral characteristics in primary-school children aged 8 to 9 years old in Italy [10]. This study supported evidence that higher Mediterranean diet adherence was related with a smaller probability of emotional undereating, whereas no such relationship was recorded for emotional overeating [10]. Thus, high Mediterranean diet adherence may be a component of a widely positive family attitude to food and children with a healthy diet may turn their negative emotions in the direction of various coping approaches, escaping the risk of emotional undereating [10]. However, the design of the above survey did not prove any causality for the considerable relationships observed. Moreover, the small participation rates and subsequently low sample size could also affect the precision of the study findings [10].

## 4. Conclusions

Emotional eating is related with psychological state (depression, anxiety), overweight/obesity and unhealthy dietary patterns. Several studies currently highlight the role of moderators including psychological state (depression, anxiety) and body weight, as well as methodological issues, such as means of provoking and measuring emotions, which could explain the equivocality regarding certain emotion and eating relationships.

When investigating emotions and eating behavior, psychological distress constituted the most frequently examined discrete emotion. Prolonged stress and depression were both related with greater food intake, with individuals eating at an increased rate throughout stressful periods [55]. Moreover, emotional eating appears to influence the kind of food eaten. The majority of food selection is characterized by more tasty and less healthy meals, while motivation to eat in order to control negative impact was related with a non-healthy eating pattern.

Van Strien et al. proposed a matched treatment strategy of obesity, and a novel pathway to more long-lasting weight decrease or maintenance of body weight [56]. However, the evaluation of the phenomenon of emotional eating is quite complex as a multilayered compilation of internal and external issues, influencing the decision to eat food and the quantity and kinds of food which will be consumed. In a meta-analysis, Evers et al. showed that for healthy individuals, obese or overweight persons, and people assessing themselves as emotional eaters negative emotions did not correlate with food intake in laboratory situations [6]. 

According to bibliography research, several well-designed clinical human studies supported an interrelationship between emotional eating and overweight/obesity, depression, anxiety/stress, and dietary patterns. However, studies presented above have a few limitations. Heterogeneity of their design, the self-reported data, the lack of balance in sex distribution, population diversities and the use of different assessment tools for emotional eating are the most common aspects that may cause bias in the generalization of the results. Moreover, the vast majority of the currently available studies had a cross-sectional design which cannot support causality, reinforcing the urgent need to conduct prospective studies. Nevertheless, there were strengths in studies with a large sample size and in those where the evaluation was carried out in person. Furthermore, there are still gaps in understanding the relationship between emotional eating and dietary patterns. In addition, to our knowledge, there is a limited number of studies that have been conducted to assess the adherence in Mediterranean diet or other healthy diets.

Overall, prevention of food use as a coping strategy is crucial among emotional eaters. Interventions such as stress management, cognitive behavioral therapy and conscious access to healthier snacks should be considered. Further clinical studies, and especially well-designed prospective studies, are strongly recommended to evaluate and confirm the potential interrelationship between emotional eating and overweight/obesity, depression, anxiety/stress, and dietary patterns.

## Figures and Tables

**Table 1:** Clinical studies evaluating the association of emotional eating with obesity.

Study Type	Study Population	Methodology	Basic Results	References
Case–control analytic study	473 participants, mean age: 32.7 (SD ± 11.4) years	Coping Strategies Inventory (CSI), Dutch Eating Behavior Questionnaire (DEBQ), ΒΜΙ categories	Overweight participants score was higher in passive coping strategies, and unhealthy eating behaviors such as emotional eating and restrained eating were recorded. Coping strategies were more likely to be associated with unhealthy eating behaviors, and these were more likely to provide and retain a high BMI.	Varela et al., 2020 [24]
Cross-sectional study	1626 adults, mean age: 30 (SD ± 11.0) years	Emotional Eating Scale (EES), ΒΜΙ categories	Emotional eating was more common in obese individuals (43.5%) compared to normal weight (33.5%) and underweight (18.4%) individuals.	Madali et al., 2021 [25]
Longitudinal study	592 adults, mean age: 45.04 (SD ± 3.9) years	DEBQ, Depressive Mood List (DML) and BMI categories	Emotional eating predicted higher increases in BMI regardless of depressive symptoms only in women.	Van Strier et al., 2016 [26]
Cross-sectional study	600 participants, mean age: 25.4 (SD ± 5.13) years	Difficulties in Emotion Regulation Scale (DERS), the Depression Anxiety and Stress Scale (DASS-21), and the Emotional Eating subscale of the Dutch Eating Behavior Questionnaire (DEBQ-EE), BMI categories	Emotional eating was related to higher BMI.	Guerrini-Usubini et al., 2023 [27]
Prospective study	39.771 adults, mean age: 49.9 (SD ± 13.7) years	Revised 21-item Three-Factor Eating Questionnaire (TFEQ-R21), the CFC questionnaire (CFC-12) and the Barratt Impulsiveness Scale (BIS-11), BMI categories	Emotional eating was positively associated with BMI.	Bénard et al., 2018 [28]
Descriptive study	298 adults, mean age: 34.08 (SD ± 9.50) years	DERS, the Positive and Negative Affect Schedule (PANAS), the Feeling of Stress Questionnaire and TFEQ-R18, BMI categories	Emotional functioning was related to BMI in adults. Snacking exhibited an indirect impact on BMI (through emotional eating).	Czepczor-Bernat et al., 2021 [29]
Cross-sectional study	605 women, mean age: 29.8 (SD ± 9.6) years	Positive–Negative Emotional Eating Scale (PNEES), Eating Disorders Assessment Scale (EDAS), and DERS, BMI categories	Negative emotional eating may be a significant risk factor for disordered eating, independently of BMI.	Sultson et al., 2019 [30]
Prospective study	3735 participants, mean age: 52.6 (SD ± 13.5) years	Depression Scale, TFEQ-R18, physical activity and night sleep duration, BMI categories	Emotional eating was one behavioral mechanism among depressive symptoms and development of obesity and abdominal obesity.	Konttinen et al., 2019 [31]
Cross-sectional study	1453 adults, mean age: 20.6 (SD ± 2.5) years	Self-Efficacy in Emotion- and Stress-Related Eating of the Eating and Appraisal Due to Emotions and Stress Questionnaire (EADES), CES-D, BMI categories	Emotional eating was associated with BMI in men as well as in women.	Lazarevich et al., 2016 [2]
Cross-sectional study	555 participants, mean age: 22.6 (SD ± 0.4) years	TFEQ-R18, BMI, percent body fat by dual-energy X-ray absorptiometry, and central obesity	Emotional eating was associated with obesity, defined by % body fat and abdominal obesity in men and women and with obesity, defined by BMI, in women.	Pacheco et al., 2021 [32]
Cross-sectional study	400 adults aged 18 to 59 years	Emotional Eating Questionnaire BMI categories	Emotional eating was more common in overweight and obese participants	Calderón-Asenjo et al., 2022 [33]
Cross-sectional study	1126 adolescents aged 15 to 20 years	Emotional Eating Questionnaire, BMI categories	Overweight and obese adolescents had a higher prevalence of negative emotional eating behavior	Skolmowska et al., 2022 [34]

**Table 2:** Clinical studies evaluating the association of emotional eating with depression.

Study Type	Study Population	Methodology	Basic Results	References
Longitudinal study	592 adults, mean age: 45.04 (SD ± 3.9) years	DEBQ, DML and BMI categories	Depressive symptoms were related to higher emotional eating. This mediation effect was independent of 5-HTTLPR genotype.	Van Strier et al., 2016 [26]
Cross-sectional study	1453 adults, mean age: 20.6 (SD ± 2.5) years	(EADES), CES-D, BMI categories	Depressive symptoms were associated with emotional eating in both sexes.	Lazarevich et al., 2016 [2]
Cross-sectional study	189 adults, mean age: 41.78 (SD± 13.61)	EES, The Emotional Appetite Questionnaire (EMAQ)Symptom Checklist-90-Revised (SCL-90), The Eating Disorders Examination Questionnaire (EDE-Q), DERS, short-form health survey (SF-12), BMI categories	Eating in response to depression (EE-D) was the kind of emotional eating most strongly associated with psychological well-being, eating disorder symptoms, and emotion regulation complications.	Braden et al., 2018 [20]
Prospective study	3735 participants, mean age: 52.6 (SD ± 13.5) years	Depression Scale, TFEQ-R18, physical activity and night sleep duration, BMI categories	Eating induced by negative emotions facilitated the positive associations with depressive symptoms.	Konttinen et al., 2019 [31]
Cross-sectional study	248 participants mean age: 25.5 (SD ± 3.8) years	Inventory of Depression and Anxiety Symptoms (IDAS), Five Factor Mindfulness Questionnaire (FFMQ), TFEQ-R18	Depression substantially interrelated with nonjudging of inner experience to predict emotional eating.	Hsu et al., 2021 [38]
Randomized controlled trial	990 overweight or obese participants, mean age: 51.7 (SD ± 13.5) years	DEBQ, 30-item Inventory of Depressive Symptomatology—Self Report (IDS-SR), semiquantitative food frequency questionnaire (FFQ)	Depression history and severity were related with more emotional and uncontrolled eating.	Paans et al., 2019 [39]
Cross-sectional study	120 obese participants, mean age: 43.13 (SD ± 13.56) years	DEBQ, Beck Depression Inventory Short Form (BDI-SF), Cognitive Emotion Regulation Questionnaire	Higher levels of emotion dysregulation were directly and strongly associated with higher levels of depression and anxiety in both (MO) (30 ≤ BMI < 40) and 60 with “severe obesity” (SO) (BMI > 40).	Willem et al., 2020 [36]
Cross-sectional study	400 adults aged 18 to 59 years	Emotional Eating Questionnaire Patient Health Questionnaire-2 (PHQ-2) scale	Depression symptoms were associated with negative emotional eating.	Calderón-Asenjo et al., 2022 [33]
Cross-sectional study	2055 participants, mean age 27.1 ± 9.52 years	Emotional Eating Questionnaire, Depression, Anxiety, Stress Scale (DASS-42)	Emotional eating was significantly correlated with perceived depression symptomatology.	Kaner et al., 2022 [40]
Cross-sectional study	506 participants, mean age: 38.59 (SD ± 11.75) years	4-part questionnaire including: Emotional Eater Questionnaire (EEQ), PSS-14	BMI was positively associated with Emotional Eater Questionnaire scores.	Barcın-Güzeldere et al., 2022 [41]

**Table 3:** Clinical studies evaluating the association of emotional eating with anxiety/stress.

Study Type	Study Population	Method	Basic Results	References
Cross—sectional study	2333 participants, mean age: 25 years	Questionnaires of perceived stress and emotional eating	Association between the emotional eating score and perceived stress.	Carpio-Arias et al., 2022 [44]
Cross—sectional study	2379 young adult women, age range: 18–19 years	Perceived Stress Scale (PSS), Nutrition Patterns Form, Life Events Scale, CES-D	Engagement in comfort eating could occur for people without severe depression symptoms to buffer the impacts of unfavorable life experiences on perceived psychological stress.	Finch et al., 2015 [45]
Cross—sectional study	600 participants, mean age: 25.4 (SD ± 5.13) years	DERS, DASS-21, DEBQ-EE, BMI categories	Substantial associations between emotion dysregulation, psychological distress, emotional eating, and BMI.	Guerrini-Usubini et al., 2023 [27]
Prospective study	43 participants (female),mean age: 19.5 (SD ± 1.3) years	Laboratory test, The Trier Social Stress Test, PSS -10, BDI, TFEQ-R18, Stress intensity, Positive and Negative Affect Schedule, Drive to eat	Higher perceived life stress increased the hyperphagic effects of stress-stimulated negative affect.	Klatzkin et al., 2019 [46]
Cross-sectional study	868 participants, mean age: 33.53 (SF ± 11.98) years	Eating Motivation Survey, the Emotional Overeating Questionnaire, COVID-19-related stress	Emotion-related predictors were associated with higher emotional overeating.	Modrzejewska et al., 2021 [47]
Cross-sectional study	254 participants, mean age: 35.82 (SD ± 11.82) years	Sociodemographic and lifestyle questionnaire, Anthropometric data, Disordered Eating Behaviors Screening Questionnaire, Coronavirus Impact Scale (CIS), DASS-21, TFEQ-R21	The change enforced by the psychosocial effect of COVID-19 lockdown on disordered eating behaviors was considerably related with psychological distress.	Ramalho et al., 2022 [48]
Cross-sectionalstudy	24,968 participants, age range: 18–70 years	Structured questions on dietary habits, emotional eating, psychological distress symptoms, and COVID-19-related worries	Emotional eating was strongly associated with psychological distress.	Bemanian et al., 2020 [49]
Cross-sectional study	400 adults aged 18 to 59 years	Emotional Eating Questionnaire, Generalized Anxiety Disorder Scale (GAD-2)	Negative emotional eating was related with the presence of anxiety symptoms.	Calderón-Asenjo et al., 2022 [33]
Cross-sectional study	2055 participants, mean age 27.1 ± 9.52 years	Emotional Eating Questionnaire, Depression, Anxiety, Stress Scale (DASS-42)	Negative emotional eating was related with the presence of anxiety and stress symptoms.	Kaner et al., 2022 [40]
Cross-sectional study	450 women, mean age 30.25 ± 10.70 years	Coronavirus Anxiety Scale, Stress Coping Styles Scale and Emotional Eating Scale	Emotional eating was associated with Coronavirus anxiety, and stress coping styles.	Güner et al., 2022 [50]

**Table 4:** Clinical studies evaluating the association of emotional eating with dietary patterns.

Study Type	Study Population	Method	Basic Results	References
Cross-sectional study	763 participants, mean age: 38 (SD ± 11.1) years	EEQ, semiquantitative FFQ	Having abdominal obesity and being an emotional or very emotional eater was clearly associated with the “Snacks and fast food” dietary pattern and adversely with adherence to the “Healthy” dietary pattern.	Betancourt-Núñez et al., 2022 [53]
Cross-sectional study	252 participants, mean age: 21.42 (SD ± 4.73) years	Adherence to the Mediterranean diet: (KIDMED) test, Alcohol Use Disorder Identification Test (AUDIT), State-Trait Anxiety Inventory (STAI), EEQ	A high percentage of individuals had an inadequate diet (20.7%) or had eating behaviors which needed improvement.	Carlos et al., 2020 [54]
Cross-sectional study	1626 adults, mean age: 30 (SD ± 11) years	EES, ΒΜΙ categories	Obese individuals raised the intake of fresh vegetables, fruits, pastries, and eggs; underweight individuals increased the intake of fresh vegetables and fruits, milk and eggs.	Madalı et al., 2021 [25]
Cross-sectional study	178 children aged 8 to 9 years	Children’s Eating Behavior Questionnaire (CEBQ), KIDMED questionnaire	A good adherence to the Mediterranean Diet was related with a smaller probability of emotional undereating.	Buja et al., 2022 [10]

## Data Availability

Not applicable.
